# Virtual neurophysiology laboratories for life science education: action potentials and voltage-/patch-clamp recordings

**DOI:** 10.1186/1471-2202-14-S1-P381

**Published:** 2013-07-08

**Authors:** Aubin Tchaptchet, Horst Schneider, Hans A Braun

**Affiliations:** 1 AG Neurodynamics, Philipps-University, Marburg, 35037, Germany; 2 DAQ-Solutions, Nehren, Germany

## 

Understanding neurophysiology is for many students a particularly difficult task, especially the interpretation of different measures their physiological meaning and their interrelations. It could be of great help if the students could do the recordings themselves, according to the didactically advantageous strategy "learning by doing". When such experiments are not possible in regular students' courses, for whatever reasons (too difficult, no resources etc.), virtual laboratories can become valuable alternatives.

During recent years, we have developed a series of virtual laboratories (Virtual Physiology), among them three neurophysiology tools. There is "SimNerv" for clinically still most relevant extracellular recordings of compound actions potentials. A second program, "SimPatch", is for whole-cell current recordings from different types of neurons, also allowing the application of channel blockers or changing ion concentrations. These programs are providing realistically appearing laboratories on the computer screen that allow the students performing physiological and pharmacological experiments almost as in real life. All stimulus and recording parameters of the virtual devices are freely adjustable and a mathematical algorithm guarantees for the appropriate reaction of the virtual preparations.

A third series of applications, "SimNeuron", has been designed in a more simple, easy to overlook form to demonstrate the relations between current-clamp, whole-cell voltage-clamp and single-channel patch-clamp recordings. This program also allows changing the model neuron's parameters to examine how altered membrane properties are reflected in the different recordings.

All these programs are designed with the major goal to train the students' understanding and know-how, although factual knowledge is a prerequisite for systematic experimentation in the virtual labs. With this respect, detailed tutorial and protocol form are provided, supplemented by additional simulations and animation of specific features.

**Figure 1:**
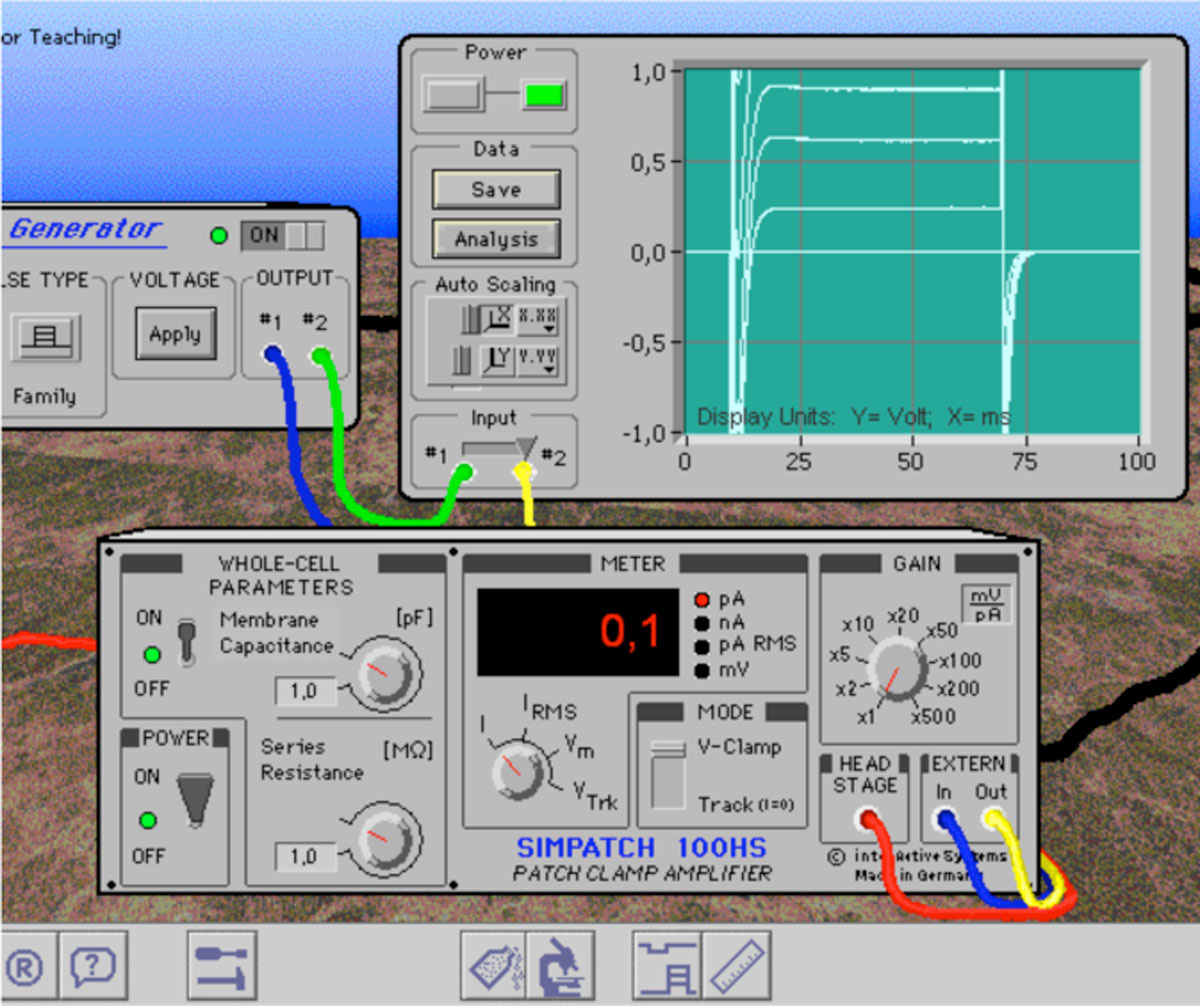
SimPatch, the Virtual Lab

**Figure 2:**
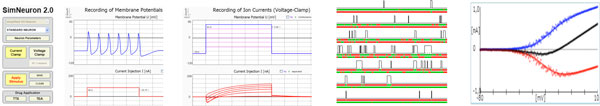
SimNeuron menu and recording examples from the current-clamp, whole-cell voltage-clamp lab, single channel patch-clamp lab (closed and open states with gating) with I-V curves for K^+^- (blue), Na^+^- (red) and unspecific cation (black) ion channels

